# Protocol for a Nested, Retrospective Study of the Australian Placental Transfusion Study Cohort

**DOI:** 10.7759/cureus.27693

**Published:** 2022-08-04

**Authors:** Ava G Tan-Koay, Sol Libesman, Martin Kluckow, Andrew W Gill, Koert de Waal, William Tarnow-Mordi, Kristy P Robledo, Anna Lene Seidler, Helen G Liley

**Affiliations:** 1 National Health and Medical Research Council (NHMRC) Clinical Trials Centre, The University of Sydney, Sydney, AUS; 2 Department of Neonatology, Royal North Shore Hospital, Sydney, AUS; 3 Northern Clinical School, The University of Sydney, Sydney, AUS; 4 Centre for Neonatal Research and Education, The University of Western Australia, Perth, AUS; 5 Department of Neonatology, John Hunter Children's Hospital, Newcastle, AUS; 6 School of Medicine and Public Health, University of Newcastle, Newcastle, AUS; 7 Newborn Medicine, Mater Mothers' Hospital, South Brisbane, AUS; 8 Mater Research, Faculty of Medicine, The University of Queensland, Brisbane, AUS

**Keywords:** newborn blood transfusion, mediation analysis, delayed cord clamping, immediate cord clamping, neonatal

## Abstract

Background

Neonates, particularly if born preterm or with congenital anomalies, are among the pediatric patients most likely to need blood transfusion. However, they are also particularly vulnerable to adverse consequences of blood transfusion. Aiming to clamp the umbilical cord for at least a minute after birth is a simple safe procedure that is being increasingly adopted worldwide, although may be associated with increased rates of polycythemia and jaundice. It may also reduce the proportion of preterm babies who need a blood transfusion. The mechanisms for this are not fully understood. Potential mechanisms could include an increased volume of blood transfusion from the placenta to the baby after birth, and an overall reduction in the severity of illness in the first weeks after birth, which could lead to fewer blood tests and greater tolerance of anemia, or enhanced erythropoiesis.

Objectives

To investigate the mechanism behind the reduced need for blood transfusions after deferral of cord clamping.

Methodology

This protocol outlines the methods and data analysis plan for a study using nested retrospective data from a large randomized trial combined with additional data collected from patient medical and pathology records. The additional data items to be collected all relate to the receipt of transfusion and the factors that affect the risk for transfusion in preterm babies. The analysis will include all randomized babies from Australia and New Zealand for whom data are available. Causal mediation analysis is planned to estimate the effects of mediators on the relationship between the timing of cord clamping and the need for blood transfusion. The analysis is designed to discern whether initial severity of illness or the magnitude of placental transfusion mediates red blood cell transfusion dependence.

Anticipated outcomes and dissemination

We expect the study will identify potential strategies for reducing blood transfusions and associated negative outcomes in preterm infants. This will be relevant to researchers, clinicians, and parents. The results will be disseminated through publications, presentations, and inclusion in evidence-based guidelines.

## Introduction

Immediate cord clamping (ICC), within a few seconds after birth, became routine in the latter half of the 20th century, as part of a tranche of medical birth-related interventions that collectively, undoubtedly improved maternal and neonatal survival and outcomes [1]. The trend to ICC (within 15-20 seconds after birth) was partly driven by some early studies suggesting that the most benefit in terms of blood volume is achieved within this time frame [2], and that deferred cord clamping (DCC) increased rates of polycythemia and jaundice [1]. It may also have been partly driven by increased rates of operative deliveries and consequent pressure to minimize surgical times, as well as the increased availability and effectiveness of neonatal resuscitation. Furthermore, ICC was proposed as a means to reduce the risk of maternal exposure to fetal blood group antigens at a time (before RhD immunoprophylaxis) when hemolytic disease of the fetus and newborn was far more common than it is now.

Formal evidence that ICC was beneficial was never sought, and recent research summarized in systematic reviews [3-6] has suggested that it may be harmful when compared with DCC for various intervals from 30 seconds until when the cord stops pulsating (defined in some studies as physiological cord clamping). ICC before the onset of breathing exposes the newborn baby to a period of significantly restricted cardiac function, whereas DCC until after the onset of breathing (which often does not occur until late in the first minute after birth) may mean that the expanding pulmonary circulation is able to fill with blood from the placenta, rather than by reverse flow across the ductus arteriosus [7]. This may improve left ventricular preload and stabilize pressures and flows in major vessels [7].

In addition, when cord clamping is deferred, babies may receive a transfusion of blood from the umbilical cord and placenta. A recent systematic review demonstrated that DCC in preterm babies improves peak hematocrit in the first week by 2.7% (95% confidence intervals (CI) 1.88-3.52) and reduced the proportion of babies receiving any subsequent blood transfusion (RD: -0.07, 95%CI -0.11 to -0.04) [6]. Some studies have found a weight increase in the first two minutes after birth when the cord is not clamped, supporting the hypothesis of placental transfusion [8]. Yet, recent evidence shows that placental transfusion may not always occur (Conference abstract: Vijayaselvi R, Abraham A, Kumar M, Kuruvilla A, Mathews J, Duley L. Measuring Umbilical Flow and Placental Transfusion for Preterm Births: Weighing Babies at 33-36 Weeks Gestation with Cord Intact. 1st Congress of Joint European Neonatal Societies; 2015).

The relative roles of cardiovascular stabilization at birth versus placental transfusion in improving outcomes have not been established. Understanding the contributions of these two mechanisms has significant implications for research and practice: for example, if the size of placental transfusion is more important, then prescribing a top-up transfusion soon after birth for babies with lower than average hemoglobin (who are known to be at higher risk of various adverse outcomes) [9] may be justified, especially for the babies for whom DCC has been precluded by maternal or fetal conditions. These include significant maternal bleeding, and monochorionic twins, where deferred cord clamping in the first twin could lead to one twin losing blood to the other. However, if it is the effects on improving cardiovascular stability in the first minutes (with consequential benefits for cardiorespiratory function and reducing severity of illness during the subsequent neonatal intensive care unit (NICU) stay), regardless of the magnitude of transfusion, then early top-up transfusion is unlikely to be helpful.

Observational studies suggest that exposure to blood transfusion itself is harmful to preterm babies, increasing the risk of adverse outcomes [10]. However, this suggestion has not been supported by the small number (to date) of randomized controlled trials of blood (red cell) transfusion thresholds [11-14]. It is unlikely to be the means by which DCC reduced deaths in the largest trial to date of deferred cord clamping in preterm babies, the Australian Placental Transfusion Study (APTS), and in the most recent systematic review on this, because neither showed a difference in rates of other adverse outcomes [6,15].

Another possibility is that it is the umbilical cord blood stem cells received by the baby are the main reason for the observed benefits to both survival and reduced requirement for later blood transfusion [16]. Umbilical cord blood has been demonstrated to be such a good contributor to hematopoiesis that it is a recognized stem cell resource for pediatric and adult hematopoietic stem cell transplant [17]. In addition, umbilical cord blood is a potential regenerative and immunomodulatory agent for a variety of clinical conditions [18], so in this case, the extent of placental transfusion would be critical to the improvement of outcomes, and transfusion with adult red cells would not suffice. There are no established methods to quantify the contribution of umbilical cord stem cells to placental transfusion. However, a larger volume of placental transfusion results in the baby receiving more nucleated cells [19], including more umbilical cord stem cells.

Discerning whether these effects (initial enhanced cardiovascular stability leading to early and sustained reduction in severity of illness or volume of placental transfusion) appear to be the main driver of improved outcomes is likely to contribute to practice change, as well as to informing the design of future research studies into methods to improve outcomes of high-risk newborn babies and reduce their transfusion dependence.

The causal mechanisms of reduced transfusion requirements found in DCC relative to ICC are yet to be resolved. The aim of the study is to address the question; In preterm infants (P) does DCC (I) compared to ICC (C) reduce dependence on red cell transfusion via enhanced cardiovascular stability (mediator 1, M1) or via an increased volume of placental transfusion (M2).

## Materials and methods

Study design

The study is a nested retrospective study, called the Transfusions in the APTS Newborns Study (TITANS) (study registration: ACTRN12620000195954), of the cohort of babies who were enrolled and randomly assigned to ICC or DCC in the Australian and New Zealand (NZ) sites for APTS (study registration: ACTRN12610000633088). This design has been developed to take advantage of the comprehensive dataset already collected for APTS, and because there is currently no suitable prospective study that could address the same research questions in such a large group of participants.

Babies had been considered eligible for APTS if obstetricians or maternal-fetal medicine specialists anticipated that delivery would occur before 30 weeks of gestation. Exclusion criteria included fetal hemolytic disease, hydrops fetalis, twin-twin transfusion, genetic syndromes, and potentially lethal malformations. Further details are available in the original APTS publication [15]. In the present TITANS analysis, we will also exclude any baby with a diagnosis of hemolytic anemia or aplastic/hypoplastic anemia.

Recruitment and data collection

There were 1401 babies enrolled for APTS from the 13 Australian and 5 NZ hospital sites [15]. APTS data was provided to the TITANS team on 31 July, 2020. It is planned to collect additional data from Australian and NZ APTS sites using a customised, secure web-based database application (REDCap) [20], which is maintained by the University of Sydney, Sydney, Australia. Data will be obtained from source documents (patient hospital records and laboratory reports) using the electronic data collection application from each study site. The individual participant data collected will correspond to the minimum data required to answer the research questions. Baby identification (ID) and other babies’ details from APTS will be used to re-identify participants and link them to hospital records. Identified data will be collected, in order to allow linkage between the data newly collected from patient records and hospital laboratories and the existing APTS dataset. The data will be checked with respect to range, internal consistency, consistency with published reports and missing items. After data cleaning and analysis, data will be stored in re-identifiable form, with each participant’s data being identified with the same study numbering system as used for the APTS study.

Objectives

**Figure 1:**
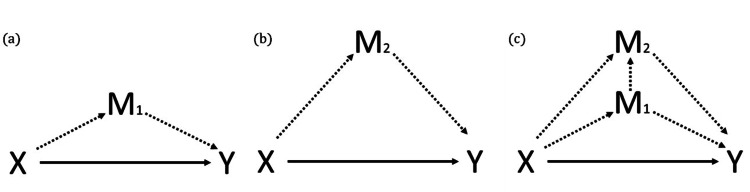
Diagrams of the possible causal mechanisms for the outcome (Y) based on the cord clamping intervention (X) (a) A single mediator model in which the intervention X (intervention – ICC or DCC) affects the outcome Y through the indirect path of the mediator M1 (placental transfusion) and via a direct path from X to Y. In this model, M1 is assumed to be independent of M2; (b) An independent mediator model in which the intervention X (intervention – ICC or DCC) affects the outcome Y through the indirect path of the mediator M2 (initial severity of illness stability) and via a direct path from X to Y. In this model, M2 is assumed to be independent of M1; (c) If M1 is not independent of M2 the assumptions of models (a) and (b) are violated and we need to instead apply a multiple mediator model in which the intervention (X) affects the outcome (Y) through both mediators (M1 and M2) and via a direct path X to Y. In model (c) it is assumed that M1 has an acyclic relationship with M2, i.e., M1 causally influences M2. Careful consideration is needed to determine the sequential ordering of this relationship, this is discussed in the data analysis plan. ICC: immediate cord clamping; DCC: deferred cord clamping Image credit: study author; concept from Imai and Yamamoto [21] and Lee et al. [22]

We will combine the data already extracted, stored and cleaned from APTS with the additional data obtained from study sites for each participating baby, to determine which factors are most influential in reducing transfusion requirements. The specific objectives are, after adjustment for prior risk factors (listed below), to determine:

1. Whether the effect of the intervention (cord clamping) on the outcome (blood transfusions) is mediated by placental transfusion (measured by hematocrit (Hct)) as seen in Figure 1 (a, c) following the causal path X → M1 → Y, where X is the intervention, ICC or DCC, Y is the outcome, mediator M1 is placental transfusion, and M2 is initial severity of illness stability

2. Whether the effect of the intervention (cord clamping) on the outcome (blood transfusions) is mediated by initial severity of illness (respiratory support, sampling line yes/no and total duration number, blood pressure, cumulative blood sample volume) as seen in Figure 1 (b, c) following the causal path X → M2 → Y

3. Whether the effect of cord clamping intervention on the outcome (blood transfusions) is driven by multiple mediators (placental transfusion and initial severity of illness) as seen in Figure 1 (c)

4. Whether cording clamping intervention (ICC or DCC) has a direct effect on the outcome after accounting for the mediators as seen in all panels of Figure 1: X → Y.

Ethical considerations

The protocol was approved by the Northern Sydney Local Health District Human Research Ethics Committee in November 2019 (Version 3.0, Reference 2019/ETH12819), the Mater Misericordiae Ltd Human Research Ethics Committee (Version 1.0, Reference HREC/MML/56247), the Mercy Health Human Research Ethics Committee (Version 2.0, Reference 2020-078), and the Southern Health and Disability Ethics Committee (Version 1.0, Reference 19/STH/195). The ethics committees have granted a waiver of consent. The study is conducted in accordance with the National Health and Medical Research Council Statement on Ethical Conduct in Research Involving Humans.

Study measures

Intervention

The intervention consisted of either immediate or delayed cord clamping (as assigned in APTS). Immediate clamping was defined as clamping the cord within 10 seconds of delivery. Delayed clamping was defined as clamping the cord at least 60 seconds after delivery, with the infant held as low as possible, below the introitus or placenta, and with no palpation of the cord. Variations in the protocol were allowed if they would aid the mother, baby, or both. If the baby was non-vigorous (heart rate <100 beats per minute, low muscle tone, or lack of breathing, or crying), clinicians were allowed to break protocol using their discretion. Cord milking was not part of the protocol for either intervention. Further details may be sourced from the original APTS publication [15].

Outcomes

The primary outcome is the proportion of babies receiving red cell transfusion (for restoration of hemoglobin or blood volume). The secondary outcomes are number of transfusions per baby, cumulative transfusion volume (mL/kg) per baby, and primary reasons for each transfusion.

Putative Mediators

M1: Indicators of placental transfusion to be assessed will be hematocrit (on admission, highest on the first day, highest in the first week collected before any postnatal transfusion).

M2: Indicators of initial severity of illness to be assessed will be cumulative blood sample volume collected throughout hospital stay (number of blood tests multiplied by hospital’s usual sample volume for each type of test), sampling line (umbilical arterial line or peripheral arterial line) - yes/no and total duration, mechanical ventilation or inspired O2, and blood pressure.

Sensitivity Analyses (For the Primary Outcome Analysis Only)

Sensitivity analyses will adjust for the following variables: gender, birth <27 weeks vs. ≥27 weeks, method of delivery (vaginal versus cesarean), intraventricular hemorrhage (IVH) (yes/no and grade III/IV yes/no), surgery for patent ductus arteriosus (PDA), necrotizing enterocolitis (NEC), and sodium in the first 24 hours of life. We will also test model assumptions relating to sequential ignorability and post-randomization confounding (discussed further in the data analysis plan).

Potential Confounders (Covariates)

The following covariates may be used for adjustment in the analysis: gestational age at randomization before birth and any oral iron supplement pre-transfusion.

Timing of Assessments

Putative mediating variables will only be analyzed if they have been measured before the outcome and will be excluded if there is not adequate time and date information available. If the multiple mediator model is applied, careful consideration of timing information will be evaluated. If there is insufficient empirical information to conclude the causal ordering of mediators (M1 causes M2), we will adjust our analytic approach (as discussed in the analysis plan) and discuss any limitations.

Data Analysis Plan

The analysis will include all babies who were initially randomized in the APTS trial for whom we were able to obtain the relevant data and be based on intention-to-treat. All statistical analyses will be conducted in R version 4.1.3 (2022-03-10; R Foundation for Statistical Computing, Vienna, Austria). Descriptive characteristics for continuous data will be presented as means or medians, as appropriate, and categorical data will be presented as frequencies and percentages.

A model-based inference approach will be applied to estimate the average causal mediation effect (ACME), average direct effect (ADE), and the average total effect as recommended [23-25]. This approach will be applied with the R “mediation” package [26]. We will initially fit two models, one model with mediation as the dependent variable and intervention as the independent variable (mediator model), and a second model with the outcome as the dependent variable, and both mediation and intervention as independent variables (outcome model). To account for the clustering of multiples, estimates will be calculated with generalized estimating equations with a compound symmetric correlation structure to account for within subject correlations. Depending on the outcome (binary, count, skew) these will be modelled with the appropriate family and link functions.

A counterfactual framework will be applied to the mediator and outcome models to simulate the values of the mediator and outcome to estimate the potential values of the mediator. This process is used to estimate the ACME, ADE, and average total effects; 95%CI will be estimated with 1000 bootstrap simulations.

We will apply single mediator models on both placental transfusion variables and initial severity of illness variables if mediators are statistically independent, as seen in Table 1. Independence will be tested using linear regression and any appropriate link functions. If both mediators are not statistically independent, we will investigate the possibility of multiple mediator models, which require an expanded framework for analysis [21]. Here we assume that initial severity of illness is causally related to placental transfusion. For this process, we will use the method developed by Imai and Yamamoto [21] to estimate the ACME and ADE. Following this, 95%CI will be estimated with 1000 bootstrap simulations. If theoretical and empirical timing data and sensitivity analyses suggest that M1 and M2 have non-causal correlation and may be affected by an unmeasured latent mediator, we will adjust our approach to estimate interventional direct and path-specific indirect effects [27,28].

**Table 1:** Overview of all mediation models * These variables will only be explored if a sufficient number of babies had both M1 and M2 measured ICC: immediate cord clamping; DCC: delayed cord clamping; Hct: hematocrit; vol: volume; n: number; mech: mechanical; vent: ventilation; O2: oxygen

Model	X - Intervention cord clamping	M_1_ - Mediator 1 placental transfusion	M_2_ - Mediator 2 initial severity of illness	Y - Outcome blood transfusions
If M_1 _is statistically independent of M_2 _after adjusting for covariates:				
1.0	ICC / DCC	Peak Hct	-	Transfusions any/vol/n
1.1	ICC / DCC	Admission Hct	-	Transfusions any/vol/n
1.2	ICC / DCC	Day 1 Hct	-	Transfusions any/vol/n
2.0	ICC / DCC	-	Mech vent or Inspired O2	Transfusions any/vol/n
2.1	ICC / DCC	-	Sampling line total duration	Transfusions any/vol/n
2.2	ICC / DCC	-	Sampling line yes/no	Transfusions any/vol/n
2.3	ICC / DCC	-	Cumulative blood sample volume	Transfusions any/vol/n
2.4	ICC / DCC	-	Blood pressure	Transfusions any/vol/n
If M_1 _is not statistically independent of M_2 _after adjusting for covariates:				
3.0	ICC / DCC	Hct peak	Mech vent or Inspired O2	Transfusions any/vol/n
3.1	ICC / DCC	Hct peak	Sampling line total duration	Transfusions any/vol/n
3.2	ICC / DCC	Hct peak	Blood pressure	Transfusions any/vol/n
3.3	ICC / DCC	remaining Hct variables*	Mech vent or Inspired O2	Transfusions any/vol/n
3.4	ICC / DCC	remaining Hct variables*	Sampling line total duration	Transfusions any/vol/n
3.5	ICC / DCC	remaining Hct variables*	Blood pressure	Transfusions any/vol/n

Sensitivity analyses have been limited to a set of biologically plausible and clinically meaningful groups that will be explored by including them for adjustment with covariates, and with the introduction of interaction terms if appropriate. Missing data will be described, reasons for missing data will be explored, and the impact of missing data on conclusions about the treatment effect on the primary outcome will also be explored where possible (e.g., using sensitivity analyses and multiple imputation techniques).

Methodological Assumptions

The causal mediation approach assumes sequential ignorability: that the treatment effect on the outcome is not confounding and that the mediator effect on the outcome is not confounded. As treatment was randomly allocated to neonates, we will assume that the treatment-mediator relationship is not confounded. However, the mediator itself has not been randomized. Thus, unknown confounders may be driving a spurious effect in the mediator-outcome relationship. We will employ additional sensitivity analyses to estimate whether any mediation effects are sensitive to the violation of the assumption of sequential ignorability. To test the possibility of unmeasured confounders we will examine the correlation between residuals in the mediator model and the outcome model. If there is no correlation this would suggest there is no unmeasured confounding, if there is correlation between the residuals, an unmeasured mediator may be affecting both the measured mediator and the outcome. We will apply the method developed by Imai et al. and Tingley et al. [23,26] that uses sensitivity analyses to evaluate if the ACME estimate is sensitive to unmeasured confounding.

Post-randomization confounders are dependent on the treatment allocated, affect both mediator and outcome, and can corrupt the mediation estimate. In the context of the present trial, it is possible that non-adherence to the intervention is a post-randomization confounder. We are analyzing our data based on intention to treat principles; however, a sensitivity analysis based on the actual time of cord clamping to assess the influence of non-adherence with the treatment protocol on our estimates may be performed.

## Results

**Figure 2:**
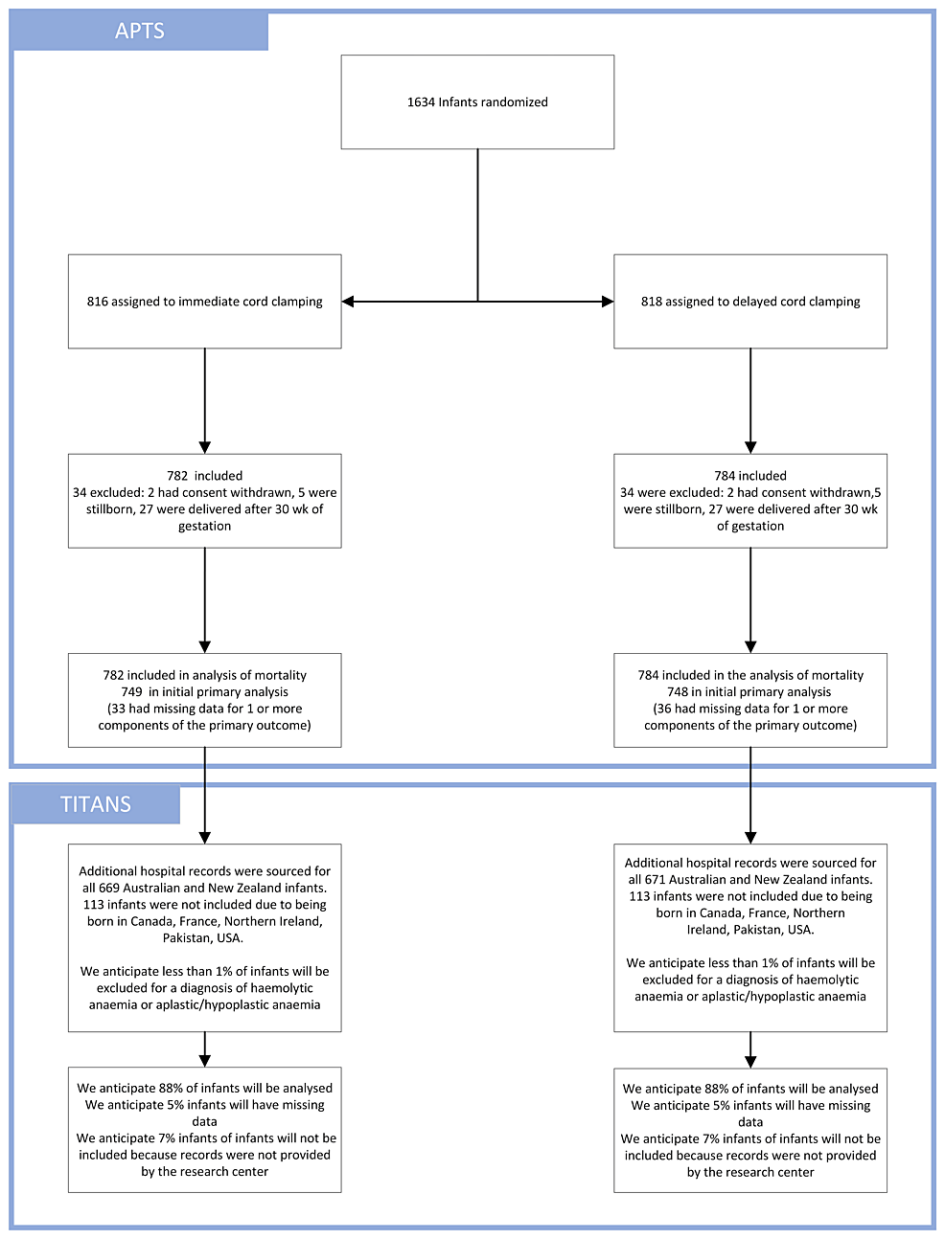
Flowchart of all infants randomized in APTS and included in TITANS. The top blue box summarizes all infants randomized and included in APTS. The second blue box summarizes the TITANS follow-up, numbers are to be inserted when data collection is complete. APTS: Australian Placental Transfusion Study; TITANS: Transfusions in the APTS Newborns Study; USA: United States of America

Figure 2 depicts the process by which infants from the APTS study were included in the TITANS study.

**Figure 3:**
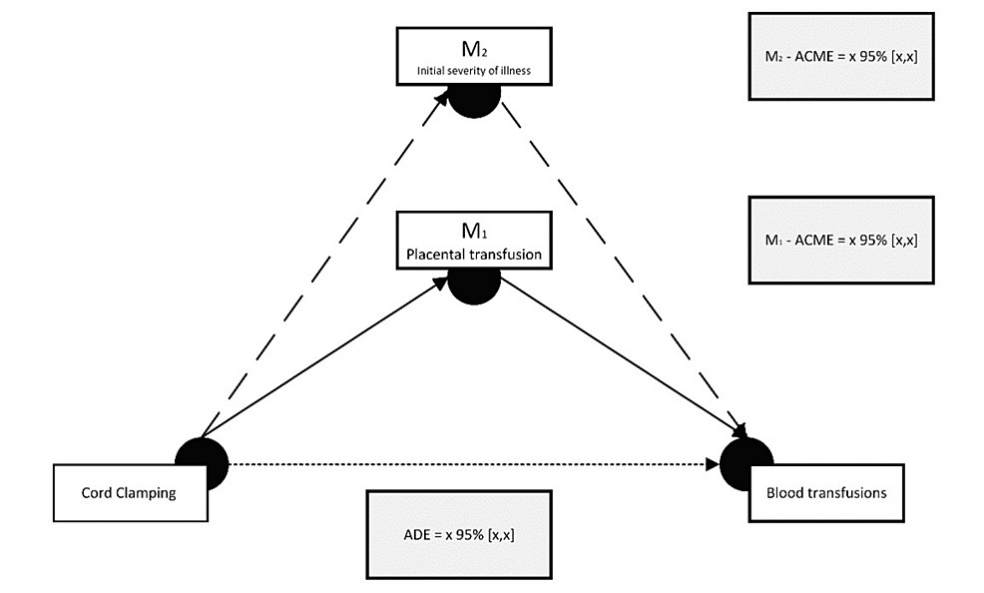
Example figure depicting the causal mediation findings if we determine multiple causally independent mediators (M1 is independent of M2) influence blood transfusions. ACME: average causal mediation effect; ADE: average direct effect

In this section, we will report the final sample size and the total number of infants data missing from the analysis. We will describe the characteristics of participants included in the mediation analysis. Both the total effect and the indirect mediation effects will be reported. All effects will be reported with 95%CI. An example template to represent our results if causal mediators are statistically independent is depicted in Figure 3. To supplement the summary of the direct and indirect effects, we will also report the relevant estimates for the specific exposure-mediator and mediator-outcome relationships. Finally, we will report the sensitivity analyses described in the data-analysis plan that assess how robust our model is to violations of its assumptions.

## Discussion

Blood transfusions of neonates have been associated with a number of serious adverse outcomes [29]. Nevertheless, there are few evidence-based methods to reduce transfusion exposure [30]. The APTS study found that DCC was associated with a statistically significant reduced need for red cell transfusions by about 10% compared to ICC [15]. However, the mechanism remains unclear. 

The study will, at a minimum, provide further information that should increase clinicians’ understanding of the pathways by which DCC (or other methods to accomplish placental transfusion) results in beneficial patient outcomes. Since one of the main barriers to implementation is lack of understanding about the mechanisms by which such a simple practice change should have such dramatic effects, this should improve adherence to recommendations to defer cord clamping for most babies, thereby reducing mortality and transfusion incidence.

By elaborating on the mechanisms, it may also provide good evidence for how other routine neonatal intensive care practices and interventions affect likelihood of needing to transfuse. Better understanding of these effects may lead to other testable hypotheses or improvements in other aspects of practice, further reducing transfusion exposure and improving other outcomes.

Potential limitations of the study include the dependence on some routinely collected clinical data, which were not collected at the time by the original study according to predefined research definitions. However, we have no reason to think that potential problems of data quality would have been influenced by study group allocation and so do not anticipate that this will be a source of bias.

## Conclusions

This study is designed to obtain and analyze additional data from the babies included in APTS to investigate the mechanism behind the reduced need for red cell transfusions. The analysis may also suggest other changes in clinical care that could reduce the need for transfusion. The outcomes of this study will directly address the research gap in relation to the role of routine use of deferred cord clamping for preterm newborn babies. As a result, the quality of guidance in relation to umbilical cord management and patient blood management in preterm babies is expected to improve.
